# Assessing REflective simulation-based e-Training on motivational interviewing among multidisciplinary healthcare practitioners [RESeT-MI]: a mixed methods pilot study

**DOI:** 10.1186/s12909-024-05711-9

**Published:** 2024-07-02

**Authors:** Angela Massouh, Nour Alayan, Mariam Shatila, Sarah Wehbeh

**Affiliations:** 1 American University of Beirut School of Nursing, Beirut, Lebanon; 2 Turpanjian College of Health Sciences American University of Armenia, Yerevan, Armenia; 3 American University of Beirut Medical Center, Beirut, Lebanon

**Keywords:** Interprofessional Education, Reflective Learning, Transformative Learning, Simulation, Blended Education

## Abstract

**Background:**

Many health science curricula have integrated behavioral modification techniques in their plans. Motivational Interviewing is one such technique. Educational interventions to promote Motivational Interviewing have had limited success. Integrating simulation-based learning in health science curricula might offer a platform whereby students can train in well controlled environments with increased authenticity, provision of standardized experiences and the capacity for immediate feedback to participants. Using motivational interviewing as an exemplar, the purpose of this study was to assess the impact of a simulation-based reflective e-training program on knowledge, attitudes, and confidence in Motivational Interviewing among healthcare practitioners from diverse healthcare disciplines. A secondary aim was to explore whether self-reflection can promote reflective learning.

**Methods:**

This was a mixed-method study design. Fifteen participants from different health disciplines were included in the quantitative phase of the study, the simulated interview, and the reflective assignments while five participated in the focus group. Pre and post tests were used to examine the effect of training on knowledge, attitudes, and confidence in Motivational Interviewing. Assessment of Motivational Interviewing Treatment Integrity [MITI] scores in a simulation-based scenario was used. A qualitative content analysis of a focus group provided a more in-depth understanding of the participants experiences. Excerpts from reflective assignments were analyzed using Transformative Learning Theory concepts.

**Results:**

A Wilcoxon test showed that the training elicited a change in confidence in performing Motivational Interviewing [Z= -2.766, *p* = 0.006], median scores increased from 29 to 34. A quarter of technical scores and half of the relational scores indicated good competence. Participants reflected content transformation through feelings of empowerment and satisfaction when they were successful in engaging and motivating clients. Process transformation was evident in reflections on how to improve core skills specifically reflective listening. Reflections on Motivational Interviewing spirit related values showed premise transformation, which may indicate attitude changes.

**Conclusion:**

A simulation-based e-training program on Motivational Interviewing represents an important educational modality for training in the health disciplines. Results of this study provide evidence supporting the integration of reflective simulation-based e-training into the education curricula of health disciplines in MI and beyond.

**Supplementary Information:**

The online version contains supplementary material available at 10.1186/s12909-024-05711-9.

## Background

Given the impact of human behavior in the pathogenesis and treatment of numerous chronic diseases, many health science curricula have integrated behavioral modification techniques as key competencies in their curricula. Yet, teaching students practical skills and techniques that they will retain and integrate into their practices is complex [1]. For that reason, it has become more critical than ever to provide diverse innovative educational interventions, use different educational methodologies to ensure information processing and retention, and provide opportunities for students to practice and communicate their views and suggestions [2].

Reflective thinking, through self-reflection or discourse with others, allows learners to gain diverse perspectives [3] and supports transformative learning [4]. This type of learning occurs when a learner is presented with information or experiences that challenge and alters their attitudes, values, and behaviors [5]. In health sciences, transformative learning helps stimulate learners’ ability to think independently, deconstruct their preconceived ideas and what they view as logical before they adhere to it as healthcare practitioners.

Motivational Interviewing [MI] is one example of a behavioral modification technique that is complex, hard to teach, and entails skills that can be difficult to acquire in traditional settings [6]. MI is a collaborative, goal directed style of communication designed to support personal motivation for and commitment to a specific goal. This is done by exploring one’s personal reasons for change within an atmosphere of acceptance and compassion and with distinct attention to the language of change [6]. Educational interventions to promote MI have had limited success [7]. Interventions that have shown success generally included two key strategies, an intensive curriculum and interactive skills practice methods [8–13]. Intensive training interventions included those that had a comprehensive curriculum, were delivered over time, focused on multiple MI skills, and used mixed teaching methods [8–10]. Another key aspect of successful educational methodologies to promote MI was the focus on experiential learning and feedback [7]. This provided learners with the opportunity to practice specific MI skills using role play, standardized patient exercises, or direct observation in clinics or through objective structured clinical examinations as well as reflect on their performance and receive direct feedback [10, 12, 14, 15].

Research has demonstrated a positive effect of MI training on confidence and knowledge but delineated such improvements to reliance on self-assessment [1] or modification of validated scales [16]. Thus, to ensure MI fidelity and standardize clinical encounters across students, an objective assessment of the integrity of MI delivered using a standardized patient is strongly recommended [17, 18]. Additionally, the use of reflection has been identified as a key element for transformative learning and as such is an important competency that educators need to facilitate in students in the context of MI [1]. Because the use of MI is relatively new in Lebanon and the region, to the best of our knowledge, no study has examined the use of MI among diverse healthcare practitioners in this region. More so, there is paucity of research on reflective simulation-based e-training on MI.

Using MI as an example, we set out to examine the impact of using reflective simulation-based e-learning to train multidisciplinary healthcare practitioners on MI with the goal of enhancing their knowledge and skills in addressing behavioral change efforts in the era of health promotion and disease prevention. Integrating reflective simulation-based e-learning in health science curricula might offer a platform whereby students can train in well controlled environments with increased authenticity, flexibility in terms of pace and learning path, and the capacity for immediate feedback to participants. The provision of standardized assessments allows for equitable experiences. Objectives of this study were to: [1] develop a reflective simulation-based e-training module [RESeT-MI] on MI for diverse healthcare practitioners; [2] assess the impact of RESeT-MI on knowledge, attitudes, and confidence; [3] measure MI fidelity and competence using a simulated patient interview; and [4] explore how self-reflection can promote transformative/reflective learning. The following questions were proposed:

- How do trainee’s self-reported *knowledge*, *attitude*, and *confidence* change following completion of the RESeT-MI e-training modules?
- Do RESeT-MI modules help trainee’s meet *MI fidelity criteria* as measured by instructor ratings?
- How does a *structured self-reflective* task promote Transformative Learning?

The significance of this study was multifaceted. Training on MI may have implications on health professionals’ contributions to behavioral change and health promotion. Additionally, the use of reflective simulation-based e-training is innovative and expected to increase student engagement and promote transformative learning [1]. Results of this study may provide evidence supporting the use of simulation-based reflection to promote transformative learning in health curricula. Qualitative evidence will provide in-depth understanding of participants’ experiences with MI, in terms of what it means to them and the challenges they encounter.

## Methods

### Study design

This study used a mixed methods pilot study design, combining a pre and post quasi-experimental approach and a qualitative assessment of reflective writings and focus group discussions. Pre and post tests were used to examine the effect of reflective simulation-based e-learning modules on knowledge, attitudes, and confidence in one’s ability to perform MI among healthcare practitioners. Quantitative assessment of students’ Motivational Interviewing Treatment Integrity [MITI] scores [Global Spirit Rating, Evocation, Collaboration, Autonomy, Direction, Empathy] in an Objective Standardized Simulation Scenario were used to assess competence in MI and treatment fidelity. A qualitative content analysis of a focus group provided a more in-depth understanding of the participants experiences. Excerpts from reflective assignments were analyzed using Mezirow’s Transformative Learning Theory.

### Participants and procedures

A convenience sample of 15 students/alumni of one of four programs at the American University of Beirut: Nursing, Psychology, Public Health, and/or Nutrition were recruited. To be eligible to participate, participants needed to meet one of the below criteria: (1) graduate or senior undergraduate nursing student; (2) practicing registered nurse; (3) graduate or senior undergraduate psychology student; (4) graduate public health student; (5) graduate or senior undergraduate nutrition student; or (6) practicing dietitian.

After securing Institutional Review Board approval, a recruitment email was sent out to the pool of eligible students at the different faculties. To avoid any undue influence, faculty members teaching students were not informed of the student’s participation in this study. They were not expected to consent the students nor give the research survey. Students were informed that neither their grades nor their evaluations will be affected due to participating or not participating in this study. Once the final sample was identified, one synchronous introductory meeting was done to discuss the study objectives. Students were informed that there will be 3 data collection points [pre-training T0, immediate post-training [T1], and 30-days post-training [T2]. Five volunteers were also asked to participate in a focus group discussion [T3]. If students verbalized their willingness to participate, they were asked to sign an informed consent before the start of this training program.

### Training modules

RESeT-MI e-training consisted of six e-learning modules [Appendix A includes the program outline] delivered between March and June 2022. Two synchronized simulation practice sessions were scheduled between modules two and three and modules 4 and 5 where students practiced MI using scenarios. Both sessions were recorded and conducted by one of the study investigators to ensure standardization. The six e-learning modules were made available at the start of the program. Participants had the option to log on to the educational platform and download the presentations, with or without voice over, and resources at their own pace within the course period. Bi-weekly optional synchronized e-consultations were scheduled via zoom and participants had access to these recordings. Forums were made available where participants can pose their questions for all to see.

The effectiveness of RESeT-MI was evaluated using: [1] pre-post evaluations of knowledge, attitudes, and confidence in using MI which will provide data essential to understand the perception and impact of the intervention on students’ subjective changes; [2] post-training instructor ratings of MI fidelity which will provide objective data regarding students’ performance and actual application of MI; and [3] focus group and reflective writings which will provide qualitative data of the training’s impact on students’ active experimentation with MI as well as on their transformative/reflective learning curve. This triangulation method of combining three measurable outcomes strengthens the quality and validity of data assessing the impact of the proposed MI training [please refer to Appendix A for the detailed modules].

### Data items

#### Knowledge and attitudes

An adapted version of the Motivational Interviewing Knowledge and Attitudes Test [MIKAT] was used to assess knowledge and attitudes of participants on MI [19]. MIKAT is a 14-item, true-false quiz that includes myths about behavior change, as well as assumptions consistent with an MI approach. The original questionnaire focuses on substance use and thus was adapted to focus on chronic illness behavioral change. It has shown sensitivity to detect change in MI consistent and inconsistent behaviour as a result of training. Cronbach’s alpha > 0.70 has been previously documented [20].

#### Confidence

Confidence in MI was measured using an eight-item survey used in a prior study [21, 22]. Confidence was scored on a 5-point Likert scale with responses ranging from 1 [Very Not Confident] to 5 [Very Confident].

#### Competence

The MITI tool was used by to assess competence in MI and treatment integrity. It evaluates component processes within MI, including engaging, focusing, evoking, and planning. The MITI has two components: global scores and behavior counts [23]. A global score on four dimensions [Cultivating Change Talk, Softening Sustain Talk, Partnership, and Empathy] requires the coder to assign a single number from a five-point scale to characterize an entire interaction. A behavior count requires the coder to tally instances of interviewer behaviors. These running tallies occur from the beginning of the segment being reviewed until the end. Typically, both the global scores and behavior counts are assessed in a random 20-minute segment [23].

#### Transformative learning

In data collection phase T2, participants were asked to take part in a 15-minute standardized and recorded actor scenario. The standardized actor, a research assistant and a healthcare practitioner with experience in behavioral modification modalities, was trained on MI by the study researchers. The actor was a silent observer who anonymously attended all the training modules and simulation sessions and then received one on one training by one of the research team members who is experienced in MI. The actor was given a detailed script that was used in all scenarios. Before being allowed to run the scenarios independently she had to successfully complete a mock interview where one of the researchers was the participant [i.e. health practitioners] and the research assistant was the client. The trained research assistant recorded the simulated scenario via Zoom video application, kept track of the 15-minute time slot, deidentified the recording, saved it as audio, and shared it with the investigators. Following this scenario, participants were asked to submit their self-assessed MITI global scores as well as reflections on their experiences, quality of their interviews, and what they perceived could have been done differently [Guidelines for Reflective Assignment are found in Appendix B].

#### Participant evaluation of the training program

Five students who accepted to take part of a focus interview were interviewed by a faculty member blinded to the study. The questions in the focus group meeting were prepared by the researchers in line with the relevant literature to determine the students’ feelings and thoughts about RESeT MI. Questions asked included: How do you evaluate your experience in using MI techniques in the standardized patient scenario? Which part of MI was easier or harder to conduct, and why? How did you perceive the person responded to your questions? How was your confidence in this process? Why did you feel confident or not? When you were conducting the interview, were you able to use the theory that you had been taught? What aligned and what didn’t, and why? As a student, what are your learning needs to improve motivational interviewing? What do you think about motivational interviewing in terms of its applicability to your future clinical practice?

### Data analysis

Quantitative data were analyzed using descriptive statistics performed using IBM SPSS Statistics version 29.0 for Windows. Qualitative data from focus groups and reflective assignment excerpts were analyzed using specific types of content analysis using transformative learning indicators based on Mezirow’s Transformative Learning Theory.

A Wilcoxon signed rank test was used to examine differences in mean scores in MI knowledge and attitudes, and confidence from pre- to post-MI training. MITI outcome measures were calculated according to formulas from the MITI coding manual as shown below. Scoring was done by one trained coder using the first 15-minute segments of the simulation recording. Two summary MITI scores included the technical global score as the average of scores on cultivating change talk and softening sustain talk and the relational global score as the average of scores on partnership and empathy. Thresholds for beginning proficiency were used for each measure.

- **Technical Global** is the average of *Cultivating Change Talk* and *Softening Sustain Talk*. Scores of 3 and 4 indicate a fair and good competence in using MI respectively.
- **Relational Global** is the average of *Partnership* and *Empathy*. A score of 3.5 indicates fair competence in using MI and a score of 4 indicates good competence.
- **Percent Complex Reflections score** is calculated as the *Complex Reflections* divided by the sum of Complex and Simple Reflections with percentages of 40 and 50 indicating fair and good competence respectively.
- **Reflection-to-Question Ratio** is the ratio of *Total Reflections* divided by *Total Questions* with 1:1 indicating fair competence in using MI and 2:1 indicating good competence.
- **Total MI-Adherent** is the sum of behavioral counts of *Seeking Collaboration*, *Affirming*, and *Emphasizing Autonomy*.
- **Total MI Non-Adherent** is the sum of behavioral counts of *Confronting* and *Persuading.*

Focus group data were grouped under the initial questions that were discussed during the session and responses were analyzed using summative content analysis. Phrases with similarities and differences in perspectives were highlighted to identify tentative patterns and compared and contrasted participants’ responses before finalizing the dominant themes and student quotes to demonstrate those ideas within each question. The two principal investigators independently undertook a content analysis of the focus group by reading and re-reading responses to each question, word by word, then undertaking memo-writing to begin formulating general impressions about students’ responses.

Transformative learning holds that the way learners interpret and reinterpret their experiences is central to making meaning and hence learning [4]. It is thus the deconstruction of learner’s prior assumptions through reflection as well as the reconstruction of their assumptions through meaning making. Learning thus involves change to meaning structures through reflection on experiences related to the *content* of the problem, the *process* of how to solve it, or the *premise* of the problem. During their reflective assignment, participants answered questions related to *what they would have done differently* and demonstrated refection on their interview performance and their learning experiences. Reflections were reviewed and analyzed using Transformative Learning Theory concepts and a framework to classify reflective practices as content, process, or premise reflections. Content reflections involve learning from present meaning and thinking back to what was done. Process reflections involve learning new meaning and considering the origin of one’s actions while premise reflections involve learning through meaning transformation and looking at a larger perspective.

The final process of data analysis included a triangulation of quantitative and qualitative results using a convergent approach [24] to assess the “fit” of data integration and the coherence of the quantitative and qualitative data.

## Results

### Participant characteristics

The mean age of the students/practitioners who participated in the descriptive part of the study was 21.6 ± 1.682 years [minimum 19 and maximum 26 years] and 80% of them were female students/practitioners. The majority [87% or 13 out of 15] of the participants had a nursing background [76.9% of the participants with a nursing background were senior undergraduate students; 15.4% were graduate students; and 7.7% were registered nurses] while 13% of the participants were practicing nutritional therapists or graduate students in psychology.

### Knowledge, attitudes and confidence in MI

About 53% of the participants had previously been exposed to MI in their curricula. As high as 80% of the participants were likely or extremely likely to use MI with persons living with chronic illness who exhibit no plan to change behavior and about 65% of the participants have used an MI approach before.

A Wilcoxon signed-rank test showed that the 6-week e-learning modules on MI for people living with chronic illness did not elicit a statistically significant change in knowledge and attitudes towards MI [Z= -1.876, *p* = 0.061]. Indeed, the median MIKAT score only changed from 8 to 9 post training. On the other hand, Wilcoxon signed-rank test showed that the e-learning modules elicited a statistically significant change in confidence in performing MI [Z= -2.766, *p* = 0.006]. In fact, the median confidence score changed from 29 to 34 post training.

### MITI scores

Means and standard deviations of the MITI outcome measures are reported in Table 1. Technical and Relational Global scores ranged from 2.5 to 4.5 [mean 3.6 ± 0.6 for the technical global and 3.6 ± 0.74 for the relational global scores]. Scores in this sample were categorized as midway between fair and good competence in MI use, with 74% of global technical scores and 47% of relational global scores indicating good clinical MI competence.

**Table 1:** MITI scores

Item	Minimum	Maximum	Mean	Standard Deviation
Change Talk	3	5	3.47	0.640
Sustain Talk	2	5	3.73	0.704
Partnership	3	5	3.8	0.862
Empathy	3	5	3.67	0.617
Technical Global	2.5	5	3.6	0.604
Relational Global	2.5	5	3.6	0.737
% Complex Reflection	0	40	13.83	14.352
R: Q Ratio	2:10	17:5	5:13	3:5
Total MI Adherent	2	13	6.66	3.039
Total MI Non-Adherent	0	6	1.933	1.751

### Focus Group

Three major themes [Program Effectiveness; Transformative Learning; and Cultural and Linguistic Lens] and seven subthemes [Engagement; Self-Paced and Balanced Guidance; Limited Opportunities for Practice; Reflections; Deconstructing Past Approaches; Language Challenges; and Cultural Barriers] emerged during the focus group discussion evaluating RESeT-MI as follows [please refer to Table 2].

**Table 2:** Focus group themes, subthemes, and quotes

Theme 1: **Program Effectiveness**	
**Sub-Themes**	**Quotes**
**Engagement** Participants thought the e-training program was able to keep them engaged despite its flexible nature due to the multiple activities and examples shared.	• “I really enjoyed the course. I think the activities and examples really kept us engaged even if it was online.”
**Self-Paced and Balanced Guidance** Participants thought course can be self-paced along with a balanced guidance from the course faculty.	• “It was useful to have the faculty guidance, but one can also take the PowerPoint and figure them out on your own. It was nice having the balance of both.” • “I believe this is a course that one can attend rather independently; there were instances of faculty guidance but at the same time we had to navigate the course to discover it.”
**Limited Opportunities for Practice** Participants still felt they needed more MI practice.	• “It would’ve been nicer to have more practical experience, although there were a lot of examples during the course and we role played a lot, but I think if there were actual patients or even if there was the opportunity to practice among our cohort it would have been nice. I feel like I know what I am doing but I lack the experience.”
Theme 2: **Transformative Learning**	
**Sub-Themes**	**Quotes**
**Reflections** Participants enjoyed being able to reflect on their attainment of the skill of MI.	• “One thing I especially liked about the delivery of the course is that it allowed us to think critically and self-reflect, because of the exercises and the interactive role plays so that is something I especially liked.” • “I became more sensitive to when people are trying to open up to me.” • “I think the application exercise was the most difficult thing. Learning how to formulate the sentences and how to spontaneously come up with responses when the patient says something you were not really expecting is challenging.”
**Deconstructing Past Approaches** Participants reflected on their past approaches in behavioral change interventions	• “After this course I realized that I might have fallen in a few traps during my clinical practice. For example, when I assume the expert role or when labeling patients. This course helped me understand that those were hindering behavioral change, affecting my relationship with the patient, and actually building up resistance instead of trust. I consciously stopped doing this now.” • “I felt like I became less radical even at home. I tried to have my dad stop smoking for as long as I can remember and I used to be very pushy but now I became more lenient, more understanding of his struggles, and celebrating with him his small victories. I am trying to celebrate these small wins, like it is very slow process, and I am less harsh on him because I know the meticulous details of this process.” • “Before taking the course when I used to talk to a patient about something I used to dominate the conversation and try to push the patient into doing something. Most of the time it would stress me out because I keep talking and keep trying to convince the patient to do something. After this course I came to believe that my role is to listen to the patient and try to help her/him reach the decision to change without me having to control the patient.”
Theme 3: **Cultural and Linguistic Lens**	
**Sub-Themes**	**Quotes**
**Language Challenges** Participants reflected on the need to have MI training using our original language and dialect and that change language is culturally sensitive.	• “I feel more comfortable speaking in English when it comes to MI, so some kind of training in Arabic would have helped. There was certain wording that we don’t really know, during the community course we tried talking to patients in Arabic and it was definitely a barrier, and I still feel that this barrier still exists here in this course.” • “I would find it difficult to do MI in Arabic and use change words translated to our dialogue.”
**Cultural Barriers** Participants alluded to the language of MI and change as easier in English than it is in Arabic. Cultural checks for Lebanon and the Arab world would help practitioners engage in MI.	• “I still feel that developing discrepancies is hard to do without unintentionally disrespecting the patient and his struggles.” • “Developing discrepancies might be perceived as belittling to one’s struggles.”

### Reflective learning

Reflections were in line with the results of the focus group session with respect to participants reflecting on the elements of the interview that generally needed attention and how these could have been improved. Reflections on self-confidence in using MI and making a difference in clients’ lives also aligned with the quantitative results of enhanced confidence. participants who had higher MITI scores had better ability to reflect on content, process, and premise. Table 3 shows the types of reflection for indicators of transformation, defined as content, process or premise in Mezirow’s Transformative Learning Theory as well as the MITI score categories. Participants reflected content transformation through feelings of empowerment and satisfaction when they were successful in engaging and motivating clients. Process transformation was evident in reflections on how to improve core MI skills specifically reflective listening. Reflections on MI spirit related values showed premise transformation, which may indicate attitude changes throughout RESeT-MI.

**Table 3:** Types of reflection and Associated MITI scores

Reflection	MITI Score	Quotes
Content Reflection Learning with present meaning [**Thinking Back**]	Good	• I attempted to ask the patient what they believe would work for them and what they believe they are capable of achieving. I also reassured the patient that the entire point of this is to find a plan that works best for the patient and that we can re-evaluate and change our plan if our primary interventions are not the most appropriate for the patient [002]. • I learned a new way of engaging with patients, one that does not solely rely on scare tactics and education, but rather on autonomy and motivation [012]. • Finding harmony between patient-centered and directive counseling is an important skill that this course helped me cultivate [019]. • I sensed that my questions were a bit directive too and cornered the patient into finding a solution for the problematic behavior that they were engaging in. However, I wanted to do what felt natural at the moment rather than follow a certain script. I worry that I may have sounded judgmental in my tone of voice while asking some questions too [019].
	Fair	• I thought that I needed to instill the motivation in the client and realized the opposite after this training [004]. • I found it satisfying that cooperation took over the conversation and the change talk and ideas for change did not end up being one sided. I was also glad that the client contributed to the session, accepted the suggestions and was willing to try out multiple modifications we agreed on [005].
Process Reflection Learning new meaning [**considering action origin**]	Good	• I should have discussed more the reasons why the person is being difficult and turn them into reasons they would want to quit [001]. • I should have explored more the patient’s values by asking them more open questions. This might help me reach a better understanding of the specific element that can push the patient to be more interested in quitting smoking and cultivate them. I should improve my skills in cultivating and softening sustain talk [001]. • I have learned that effective change can only be achieved when the patient wants to change and when our change interventions are client centered [002]. • I would spend more time reflecting on what the patient is saying. I would also give more importance to summarizing what is being said throughout the interview to ensure that both the patient and I are still on the same page [002]. • I could have inquired more about their current situation so that they would be less passive and even more engaged in our interaction. I could have also asked fewer questions and allowed the patient more time to talk so that I could get a deeper understanding of their current situation and help tailor a possibly more suitable or reasonable plan for change alongside them [019].
	Fair	• I think I should have given the client more suggestions regarding how to manage his stress and not only random solutions. The client was very clear telling me that all of his health problems and smoking are because of stress, and I should have addressed that directly [006].
Premise Reflection Learning through meaning transformation [**looking at a larger perspective**]	Good	• Motivational interviewing is a great approach because it helps the patient reflect on their values and how their behavior embodies their own short and long-term goals in life [001]. • I have learned that my job is to listen to the patient, reflect, and guide the patient through establishing a change plan best tailored to the patient’s needs [002]. • Assessment of what the patient already knows is a must before doing education [008]. • I learned that avoiding judgment and embracing the human being in front of us as a partner in the history of our evolution leading up to the moment of our interaction helps to induce a deeper sense of empathy and fosters collaboration [019]. • Small changes, accumulated, make for lasting and life-changing outcomes [019].
	Fair	• It is important to compromise and take small steps to reach the desired goal [005].

## Discussion

The present study examined a 6-module reflective simulation-based e-training on MI for people living with chronic illness. The world of simulation is a rather new modality in Lebanon and the region and so is doing it virtually. To our knowledge, our study is the first study to assess the impact of this relatively new educational modality. Moreso, exploring the effectiveness of self-reflection as an instructional strategy remains an untapped research area in the region. This study utilized multiple sources of data to evaluate RESeT-MI, most of which showed promising results. Participants expressed high satisfaction with the training but suggested the need for further faculty guidance and more practice time for skill improvement.

Pre- and post-test results showed a significant impact of RESeT-MI on the participants’ self-reported confidence related to MI use, although changes in knowledge and attitudes were not statistically significant. However, instructor ratings using MITI guidelines showed fair to good competence providing preliminary evidence on knowledge and competence gains. Some MI competencies, such as the ratio of reflections to questions, were not attained by the participants in this pilot study, an expected finding given the limited hours of practice. MITI training is usually intensive requiring extensive hours of practice; one study investigating MI fidelity using MITI recommended a minimum of two hours of training a day over four weeks with a total of 40 hours [25]. Previous studies report a wide range of training hours with inconsistent outcomes [26, 27]. Nevertheless, this study showed that 15 hours of MI training enhanced participant confidence by 17.25%, adding to the literature a potential range of hours needed to have a significant impact of training on confidence. A previous study using shorter trainings [3.5 hours] reported lower effect size [28]. Quantitative results corroborated with qualitative results which revealed that students genuinely benefited from this experience and repeatedly reported improvements in confidence related to MI use. In addition, qualitative results expanded on the quantitative results and provided an in-depth understanding of the learning process throughout the RESeT-MI in terms of content, process, and premise of transformative learning, providing additional evidence on knowledge and new evidence on attitude gains. Our findings show beginning skills of transformative learning when participants noted their increased aptitude to learn with present meaning by reflecting on their past experiences, learn with new meaning by evaluating their current experience, and finally learning through meaning transformation and reflecting on what they would do differently in the future. To our knowledge, this is the first study to link content, process, and premise of reflection with associated MITI scores. We were able to show that those participants with higher MITI scores showed higher level of reflection.

The use of a cohort of participants coming from diverse healthcare disciplines in RESeT-MI is innovative and adds to the trending direction in health and medical education [29]. Collectively, multiple sources of data from this study provided preliminary evidence on multifaceted knowledge, skills, and attitude gains in and towards MI use.

This study is especially important because of the evidence it provides supporting the integration of MI training in health curricula. MI-related practices and knowledge are limited among healthcare practitioners while the need to adopt MI approaches in clinical care is evident internationally [30]. In the context of Lebanon, a previous primary care study showed that 76% of practicing nurses and physicians lacked MI-related knowledge and practice but more than 92% were willing and ready to learn MI techniques and potentially integrate it in their clinical practice [31]. Therefore, providing RESeT-MI or similar MI trainings in higher education health curricula is timely and valuable. Future research is needed to further examine the long-term impact of such trainings on patient outcomes.

### Strengths and limitations

Findings of this research should be interpreted in light of its limitations. This pilot study included a small sample size, did not include a control group, and relied partially on self-report data. Nevertheless, the use of triangulation and convergent mixed methods approach in evaluating the effect of this MI simulation-based e-training on knowledge, confidence, and competence in MI enhanced the robustness of the analysis. The use of a trained actor with realistic scenarios, despite a virtual environment with no face-to-face interaction, most probably helped echo the clinical reality of behaviour change. It is critical to note that while the use of virtual platforms limits non-verbal communication, these offer easier accessibility and reach as well as scalability for training purposes. Additionally, MITI scoring allowed for an objective quantification of knowledge and skill gains after RESeT-MI although these were not statistically significant in the pre-post MIKAT evaluation, likely due to the limited sample size. Finally, the study quantified participants’ self-rated performance using MITI global scores but did validate their scoring against that of experts because the focus was on participants ability to reflect on their ability to use the MI skills they had learned rather than on the quality of the MI interview.

## Conclusion

MI represents an important continuing area of opportunity for training and practice. In this study, we assessed the effect of a simulation-based reflective e-training module on knowledge, attitudes, and confidence in MI, objectively measured MI fidelity using a simulated patient interview; and explored whether self-reflection can promote transformative/reflective learning. We were able to provide evidence that, although some found it challenging, but reflecting on one’s performance in terms of acquisition of MI skills and learning through meaning transformation and looking at a larger perspective provides a deep transformative learning experience. This provides evidence supporting the integration of reflective simulation-based e-training into the health sciences education curriculum in MI and beyond.

### Electronic supplementary material

Below is the link to the electronic supplementary material.

Supplementary Material 1

Supplementary Material 2

Supplementary Material 3

Supplementary Material 4

## Data Availability

The datasets used and/or analyzed during the current study are available from the corresponding author on reasonable request.

## Abbreviations

MI: Motivational Interviewing
RESeT-MI: Reflective E-Training Module on Motivational Interviewing for Interprofessional Health Teams
MITI: Motivational Interviewing Treatment Integrity
MIKAT: Motivational Interviewing Knowledge and Attitudes Test

## References

[1] Schoo AM, Lawn S, Rudnik E, Litt JC. Teaching health science students foundation motivational interviewing skills: use of motivational interviewing treatment integrity and self-reflection to approach transformative learning. BMC Med Educ. 2015;15. 10.1186/s12909-015-0512-1 PMC4687369 26689193

[2] Hung MSY, Lam SKK, Chow MCM (2020). Nursing students’ experiences and perceptions of learner-centred education in a disaster nursing course: a qualitative study. Nurse Educ Pract.

[3] McAllister MOF, Downer T, Lyons M, Pelly F, Barr N (2013). Evaluating STAR - a transformative learning framework: interdisciplinary action research in health training. Educ Action Res.

[4] Mezirow J (2000). Learning as Transformation: critical perspectives on a theory in Progress.

[5] Renigere R (2014). Transformative learning in the discipline of nursing. Am J Educ Res.

[6] Miller WR, Rollnick S. Motivational Interviewing: Helping People Change. Third ed. New York:: The Guilford Press; 2013 2013.

[7] Dunhill D, Schmidt S, Klein R (2014). Motivational interviewing interventions in graduate medical education: a systematic review of the evidence. J Grad Med Educ.

[8] Abramowitz SA, Flattery D, Franses K, Berry L (2010). Linking a motivational interviewing curriculum to the chronic care model. J Gen Intern Med.

[9] Triana AC, Olson MM, Trevino DB (2012). A new paradigm for teaching behavior change: implications for residency training in family medicine and psychiatry. BMC Med Educ.

[10] Childers JWBJ, Kraemer KL, Cluss PA, Spagnoletti CL, Gonzaga AM (2012). Giving residents tools to talk about behavior change: a motivational interviewing curriculum description and evaluation. Patient Educ Couns.

[11] Lozano P, McPhillips HA, Hartzler B, Robertson AS, Runkle C, Scholz KA (2010). Randomized trial of teaching brief motivational interviewing to pediatric trainees to promote healthy behaviors in families. Arch Pediatr Adolesc Med.

[12] Burton AM, Agne AA, Lehr SM, Davis NJ, Willett LL, Cherrington AL (2011). Training residents in obesity counseling: incorporating principles of motivational interviewing to enhance patient centeredness. J Grad Med Educ.

[13] Yu GC, Beresford R (2010). Implementation of a chronic illness model for diabetes care in a family medicine residency program. J Gen Intern Med.

[14] Cole B, Clark DC, Seale JP, Shellenberger S, Lyme A, Johnson JA (2012). Reinventing the reel: an innovative approach to resident skill-building in motivational interviewing for brief intervention. Subst Abus.

[15] Forsberg L, Forsberg LG, Lindqvist H, Helgason AR (2010). Clinician acquisition and retention of motivational interviewing skills: a two-and-a-half-year exploratory study. Subst Abuse Treat Prev Policy.

[16] Kaltman S, WinklerPrins V, Serrano A, Talisman N (2015). Enhancing motivational interviewing training in a family medicine clerkship. Teach Learn Med.

[17] Baer JS, Rosengren DB, Dunn CW, Wells EA, Ogle RL, Hartzler B (2004). An evaluation of workshop training in motivational interviewing for addiction and mental health clinicians. Drug Alcohol Depend.

[18] Madson MB, Campbell TC (2006). Measures of fidelity in motivational enhancement: a systematic review. J Subst Abuse Treat.

[19] Leffingwell TR (2006). Motivational interviewing knowledge and attitudes test (MIKAT) for evaluation of training outcomes. MINUET.

[20] Madson MB, Schumacher JA, Noble JJ, Bonnell MA (2013). Teaching motivational interviewing to undergraduates: evaluation of three approaches. Teach Psychol.

[21] Fortune J, Breckon J, Norris M, Eva G, Frater T (2019). Motivational interviewing training for physiotherapy and occupational therapy students: Effect on confidence, knowledge and skills. Patient Educ Couns.

[22] Poirier M, Clark K, Cerhan MM, Pruthi JH, Geda S, Dale YE. LC. Teaching Motvational Interviewing to First Year Medical Students to Improve Counseling Skills in Health behavior Change. Mayo Clin Proc. 2004;79. 10.4065/79.3.327 15008606

[23] Moyers TB, Rowell LN, Manuel JK, Ernst D, Houck JM (2016). The Motivational Interviewing Treatment Integrity Code (MITI 4): Rationale, preliminary reliability and validity. J Subst Abuse Treat.

[24] Fetters MD, Curry LA, Creswell JW (2013). Achieving integration in mixed methods designs-principles and practices. Health Serv Res.

[25] Kramer Schmidt L, Andersen K, Nielsen AS, Moyers TB (2019). Lessons learned from measuring fidelity with the Motivational Interviewing Treatment Integrity code (MITI 4). J Subst Abuse Treat.

[26] Bremner MN, Maguire MBR, Keen D, Blake BJ, Santa H, Nowalk A (2020). Implementation and evaluation of SBIRT training in a Community Health nursing course. Public Health Nurs.

[27] Acquavita SP, Richardson GB, Smith R, Van Loon RA, Brehm B, Kim K (2021). Outcomes of an interprofessional SBIRT training program: knowledge attainment and perceived competence for practice. Substance Abuse.

[28] Gainey S, Muzzy W, Dooley M, Lauerer J, Pelic C, Rheingold AA (2022). Outcomes and lessons learned from an interprofessional student training program in screening, brief intervention, and Referral to Treatment (SBIRT) at an academic health sciences center. Nurse Educ Today.

[29] Oxlad M, Turnbull D, Skinner V, Lekkas D (2021). Undergraduate psychology and dental students’ perceptions of interprofessional learning when using motivational interviewing to encourage health behaviour change: a mixed methods study. Australian Psychol.

[30] Lindsey AC, Janich N, Macchi CR, Cordes CC, Mendoza NS, Vazquez E (2021). Testing a screening, brief intervention, and Referral to Treatment (SBIRT) interdisciplinary training program model for higher education systems. Families Syst Health.

[31] Alayan N, Naal H, Makhoul M, Avedissian T, Assaf G, Talih F et al. Primary care screening, brief intervention, and referral to treatment for adolescent substance use in Lebanon: A National cross-sectional study. Subst Abuse: Res Treat. 2021;15. 10.1177/1178221821994608 PMC7989113 33814913

