# Supplementary material for: Assessing REflective simulation-based e-Training on motivational interviewing among multidisciplinary healthcare practitioners [RESeT-MI]: a mixed methods pilot study
Source: BMC Med Educ. 2024 Jul 2;24:711. doi: 10.1186/s12909-024-05711-9 (PMC11218146; doi:10.1186/s12909-024-05711-9)
Supplement: Supplementary file 3 — Supplementary Material 3 [file 12909_2024_5711_MOESM3_ESM.docx]

# Appendix A. Modules Outline


| **Pre-Training** | | | |
| --- | --- | --- | --- |
| **Week** | | **Topic** | **Duration** |
| Week 0 | **T0: Pretraining** | Questionnaires: Motivational Interviewing Knowledge and Attitudes [MIKAT] and Confidence in Motivational Interviewing | 30 minutes |
| **Simulation Based e-Training Program Modules** [14 weeks] | | | |
| **Week** | **Module** | **Topic** | **Duration** |
| Weeks 1 and 2 | **I:** Spirit of Motivational Interviewing | Conversations About Change | 30 minutes |
|  |  | Spirit of Motivational Interviewing | 45 minutes |
|  |  | Method of Motivational Interviewing | 45 minutes |
| Weeks 3 and 4 | **II:** Engaging: The Relational Foundation | Engagement and Disengagement | 30 minutes |
|  |  | Listening: Exploring Values and Goals | 45 minutes |
|  |  | OARS: Client-Centered Counseling Skills | 45 minutes |
| **Week 5** | **Synchronized Simulation** | **OARS Training with Scenarios and Reflection** | **90 minutes** |
| Weeks 6 and 7 | **III:** Focusing and Evoking | Focusing: Why Focus? And Exchanging Information | 60 minutes |
|  |  | Exploring Ambivalence | 60 minutes |
| Weeks 8 and 9 | **IV:** Evoking | Change versus Sustain Talk | 60 minutes |
|  |  | Evoking a Person’s Own Motivation | 60 minutes |
| **Week 10** | **Synchronized Simulation** | **Rolling with Sustain Talk and Resistance** | **90 minutes** |
| Weeks 11 and 12 | **V:** Planning | Developing a Change Plan | 60 minutes |
|  |  | Consolidating Commitment and Supporting Change | 60 minutes |
| Weeks 13 and 14 | **VI:** Motivational Conversations | Research Evidence | 60 minutes |
|  |  | Motivational Interviewing Treatment Integrity Code | 60 minutes |
| **Post-Training** | | | |
| **Week** | | **Topic** | **Duration** |
| Week 15 | **T1: Post Training** | Questionnaires: Motivational Interviewing Knowledge and Attitudes [MIKAT] and Confidence in Motivational Interviewing | 30 minutes |
| Weeks 16 and 17 | **T2: OSCE** | Motivational Interviewing Treatment Integrity Coding [Global scores by participants and detailed coding by researchers] | 30 minutes |
|  | **T2: Reflection** | Reflective Practice Assignment | 60 minutes |
| Week 18 | **T3: Focus Group** | Optional for 5 participants | 60 minutes |
