# Supplementary material for: Assessing REflective simulation-based e-Training on motivational interviewing among multidisciplinary healthcare practitioners [RESeT-MI]: a mixed methods pilot study
Source: BMC Med Educ. 2024 Jul 2;24:711. doi: 10.1186/s12909-024-05711-9 (PMC11218146; doi:10.1186/s12909-024-05711-9)
Supplement: Supplementary file 4 — Supplementary Material 4 [file 12909_2024_5711_MOESM4_ESM.docx]

# Appendix B. Guidelines for Reflective Assignment

**Using Motivational Interviewing Treatment Integrity Coding to Evaluate an MI Encounter with a Standardized Patient**

**Guidelines**

1. Students will be sent instructions via email 48 hours prior to the zoom call.
2. On the zoom call, student can choose the topic of the interview. Two options are available:
   1. A patient with uncontrolled diabetes who is Obese and has sedentary lifestyle.
   2. A patient with a cardiovascular disease who is a Heavy Smoker.
3. The standardized patient [which will be played by a Trained Research Assistant] has **low Confidence** and **high Conviction.**
4. Students will conduct an interview that ranges between 10 to 15 minutes **max.**
5. The Research Assistant will record the interview.
6. The Research Assistant will send the student an email immediately after the OSCE with instructions for the reflective assignment.

| **Scenario 1** |
| --- |
| History provided for the student:Chief Concern: 60-year-old female patient who is coming for lifestyle improvement for diabetes and obesity.History of Present Illness:  1. You have seen this patient before. 2. Last visit you advised the patient to think about daily exercise and she is now back to discuss. 3. Patient was recently hospitalized for a lower extremity cellulitis. 4. **Past Medical History:** Diabetes + Thyroid + Obesity + Hyperlipidemia + Heart Disease. Mother has hypertension and heart disease, and father has diabetes. 5. **Hospitalizations:** Recent lower extremity cellulitis where she was admitted for 4 days of antibiotics. 6. **Medications:** Novonorm 1 mg orally three times daily [Antidiabetic] + Trajenta 1 tablet orally twice daily [Antidiabetic] + Aspirin 81 mg daily [Prevention of Blood Clotting]. 7. **Social History:**  - Smokes 1 Pack per day for last 30 years. - Drinks a glass of wine with meals. - No drugs. - Sedentary lifestyle.  History given to the Standardized Patient:  1. You are aware that your recent hospitalization was because of Diabetes and obesity, and it is directly caused by poor physical activity. 2. You enjoy your sedentary life and watching TV, it relieves stress for you, and you have not thought seriously about a new lifestyle before now. 3. You know sedentary life is bad for your health, and you are thinking about changing and imparting on a healthy diet. |
| **Scenario 2** |
| History provided for the student:  1. **Chief Concern**: 40 year old male patient wants to discuss quitting smoking 2. **History of Present Illness:**    1. You have seen this patient before.    2. Last visit you advised the patient to think about quitting smoking and he is now back to discuss.    3. Patient was recently hospitalized for COPD exacerbation caused by smoking.    4. **Past Medical History:** Hyperlipidemia and Heart disease: cardiac catheterization last year and managed with medications. He also has COPD. Mother has hypertension and heart disease and father has diabetes.    5. **Hospitalizations:** Recent COPD exacerbation and cardiac catheterization and cardiac stenting in 2020.    6. **Medications:** lisinopril 10 mg daily [Antihypertensive]; atorvastatin 10 mg daily [Lipid Control]; and Aspirin 81 mg daily [Prevention Of Blood Clotting]. 3. **Social History:**     1. Smokes 1 Pack per day for last 30 years.    2. Drinks a glass of wine with meals.    3. No drugs.  History given to the Standardized Patient:  1. You are aware that your recent hospitalization was because of an illness [COPD] that is directly caused by cigarette smoking. 2. You enjoy smoking, it relieves stress for you, and you have not thought seriously about quitting before now. 3. You know smoking is bad for your health, and you are thinking about quitting. |
